# Targeting MHC-I molecules for cancer: function, mechanism, and therapeutic prospects

**DOI:** 10.1186/s12943-023-01899-4

**Published:** 2023-12-02

**Authors:** Xiangyu Wu, Tianhang Li, Rui Jiang, Xin Yang, Hongqian Guo, Rong Yang

**Affiliations:** 1 Department of Urology, Nanjing Drum Tower Hospital, Affiliated Hospital of Medical School, Nanjing University, Nanjing, China; 2 Department of Urology, Zhongda Hospital, Southeast University, Nanjing, China; 3 Surgical Research Center, Institute of Urology, Southeast University Medical School, Nanjing, China; 4 The School of Basic Medical Sciences, Fujian Medical University, Fuzhou, China

**Keywords:** MHC-I, Cancer immunotherapy, TME, B2M, Antigen presentation, Cancer immune evasion

## Abstract

The molecules of Major histocompatibility class I (MHC-I) load peptides and present them on the cell surface, which provided the immune system with the signal to detect and eliminate the infected or cancerous cells. In the context of cancer, owing to the crucial immune-regulatory roles played by MHC-I molecules, the abnormal modulation of MHC-I expression and function could be hijacked by tumor cells to escape the immune surveillance and attack, thereby promoting tumoral progression and impairing the efficacy of cancer immunotherapy. Here we reviewed and discussed the recent studies and discoveries related to the MHC-I molecules and their multidirectional functions in the development of cancer, mainly focusing on the interactions between MHC-I and the multiple participators in the tumor microenvironment and highlighting the significance of targeting MHC-I for optimizing the efficacy of cancer immunotherapy and a deeper understanding of the dynamic nature and functioning mechanism of MHC-I in cancer.

**Supplementary Information:**

The online version contains supplementary material available at 10.1186/s12943-023-01899-4.

## Introduction

The cells from jawed vertebrates need to provide the immune system with the physiological status information, thus inducing necessary elimination in face of infection and magnificent transformation or maintaining the immune tolerance under normal conditions [1, 2]. This process is dominated by the molecules of Major histocompatibility class I (MHC-I) complex, which presents the peptides on the surface of the cell. The MHC-I complex displays the structure of heterodimers, consisting the polymorphic heavy chain and the light chain β2-microglobulin (B2M) [3–5]. After presenting the peptides on the cell surface, the MHC-I complexes would get scanned by the T-cell receptors (TCRs), allowing the CD8^+^ T cells to recognize the antigenic peptides and then clean the cancerous or infected cells [6]. Obviously, this process serves as the primary step and a fundamental basis for the anti-cancer immunity and accordingly, any interference or abnormal regulation during this process may be hijacked by tumor cells to escape the immune surveillance and elimination.

Recent years witnessed great advances made in cancer immunotherapy, especially immune-checkpoint blockade (ICB), cancer vaccines, and chimeric antigen receptor (CAR) T-cells, striving to reinvigorate the T cell-mediated anti-cancer immunity to kill the cancer cells. Although significant survival benefits have been brought to a wide range of cancer patients, great difficulty and barrier in the treatment still exist, including the innate and adaptive immune resistance, complex tumor immune microenvironment, great individual difference, and the difficulty in predicting the immunotherapy effect. These challenges still seriously limited the clinical application of cancer immunotherapy.

As we mentioned before, MHC-I-mediated antigenic peptides presentation pathway is the predominate initiative factor for the anti-cancer immunity. Therefore, the importance of the MHC-I modulation in cancer immune evasion has been emphasized in recent years and a range of studies have reported that the loss or downregulation of MHC-I is a major mechanism of cancer immune evasion by blocking the surface presentation of tumor-associated antigens, thus suppressing the cytotoxicity of CD8^+^ T cells and impairing the adaptive immune response [7, 8]. Nevertheless, in addition to this obvious logical mechanism, multiple studies also uncovered a range of non-canonical biological functions of MHC-I molecules in cancer, posing a totally different direction in multiple aspects, including the partner immune cell subtypes, immune functioning mechanisms, and the interactive relationships with the tumor microenvironment. Thus, we came to realize that MHC-I molecules could participate in the tumorigenesis through multiple pathways. Meanwhile, the emerging newly-identified roles played by MHC-I molecules in the tumor microenvironment pose new questions and challenges to us. Firstly, since the MHC-I could engage in the immune response regulation of various immune cells, are these processes carried out simultaneously and independently or coupling with each other? Secondly, what up-stream factors determine or influence the direction of the MHC-I molecule-mediated tumor immunity and how the positive and negative immune effects caused by MHC-I achieve the mutual balance and transformation? Moreover, how can we utilize the mechanism of MHC-I dysfunction in anti-cancer immunity to develop more effective diagnostic and therapeutic approaches to battling against cancer immune evasion and invigorating the cancer immunotherapy efficacy? To answer these questions, we are required to comprehensively review the key studies and related literature of MHC-I in cancer context and further clarify the complex networks centered on MHC-I in the tumor microenvironment. As the first step, we need to recognize and understand the structural basis of MHC-I.

## The structure and construction of MHC-I molecules

As the material basis of adaptive immune system, the evolutionary inception of MHC complexes (MHC-I and MHC-II) dates back about 500 million years ago [9]. MHC-I molecules are commonly located on the cell surface of the nucleated cells, forming the trimeric complexes composed of a heavy chain, an invariant light chain B2M. The heavy chains are lined with the cell membrane and the domains distant to the membrane, including α1 and α2, form a groove structure for the peptide binding. The heavy chains of MHC-I are known as Human Leukocyte Antigen (HLA), which are genetically encoded by HLA-A, HLA-B, and HLA-C as the classical MHC-I genes and HLA-E, HLA-F, and HLA-G as the non-classical MHC-I genes. High polymorphism exists within the MHC-I genes results in different allelic variants of the MHC-I molecules, thus ensuring the great diversity of the bound peptide ligands and the uniqueness of the distinct presented peptide repertoires [10]. Also, the binding of the peptide endows the MHC-I complex with stabilization. Once the MHC-I molecule is loaded with the peptides, the MHC-I complex will become peptide/MHC-I (pMHC-I). The peptide displayed determines how CD8^+^ T cells will treat the pMHC-I expressed cells. When the peptides loaded by MHC-I are “non-self” with abnormally altered structures, the CD8^+^ T cells would be activated following the antigen recognition and then induce the immune killing to the aberrantly antigen-exposed cells. Meanwhile, for the pMHC-I complexes with “self” peptides, tonic signals would be released for the survival of the naïve CD8^+^ T cells [11].

The pathway of the assembly and construction of pMHC-I begins in the endoplasmic reticulum (ER). Firstly, both the classical and non-classical neoantigens would be packaged into the proteosomes in the cytosol to get trimmed into neoantigen peptides. Then the Transporter associated with Antigen Processing (TAP) will transfer the peptides to the ER, where the peptides will be further trimmed into smaller and optimized sizes (8–10 amino acids) through the aminopeptidase function of the ER aminopeptidases (ERAP)1 and ERAP2. Calnexin, a molecular chaperone of MHC-I, plays a fundamental role in the folding and assembly MHC-I heavy chain, forming the partially folded MHC-I. Afterwards, the peptide-loading complex, constructed by the TAP subunits TAP1 and TAP2, calreticulin, ERp57, and tapasin, will get the peptide loaded on the MHC-I. Subsequently, the pMHC-I molecules are transferred to the plasma membrane to be presented to the CD8^+^ T cells. Any defect in the production or function of the components of MHC-I, such as the HLA heavy chains, B2M light chains, or the peptides production and loading complex subunits, etc., will interfere the normal presentation of MHC-I molecules on the cell surface and subsequently affect the immune recognition and response of CD8^+^ T cells.

## Regulation of MHC-I expression

As an immune protein complex, the expression of MHC-I molecule is modulated by multiple mechanisms. Diverse levels could be utilized to regulate the expression of MHC-I, which provides a finer and more complex regulatory network. At the level of genetic transcription, the major regulator for the MHC-I molecule genes is Nucleotide-binding oligomerization domain-Like Receptor family Caspase recruitment domain containing 5 (NLRC5), also known as the Class I Transactivator (CITA). By binding with RFX5 and RFXAP, NLRC5 forms a CITA enhanceosome complex to promote the expression of MHC-I molecules as a transcriptional activator. As a member of leucine-rich containing proteins (NLR) family, NLRC5 also represents a major sensor for recognizing endogenous and exogenous stress and microbial infection to boost innate and adaptive immunity. The classical cellular functions of NLRC5 have been nicely reviewed and summarized by a range of studies [12]. As an upstream factor, IFN molecules (IFN-α, β, and γ) increase the expression of MHC-I through the JAK/STAT pathway, which subsequently activate the transcriptional functions of NF-κB and IRF to bind to the Enhancer A region and the interferon-stimulated response element (ISRE) in the promoter region of MHC-I and thus promote the expression of MHC-I [13, 14]. Notably, a range of studies also revealed that NLRC5 could also in turn modulate the IFN responses in different ways, which suggested that the interactive relationship between IFN and NLRC5 might form a feedback loop to regulate the expression of MHC-I. More recently, Chen et al. utilized a specific pMHC-I-guided CRISPR-Cas9 screening method to identify crucial MHC-I regulators and found that a key inhibitory molecular SUSD6/TMEM127/WWP2 axis, in which SUSD6, TMEM127, and MHC-I forms a trimolecular complex to recruit WWP2 for MHC-I ubiquitination and lysosomal degradation. This process resulted in the decrease of MHC-I expression and impaired anticancer immunity, which subsequently shortens the cancer patients’ survival periods [15]. This newly-identified inhibitory pathway may be a novel therapeutic target for reinvigoration of CD8^+^ T cells.

Moreover, the expression of MHC-I is also regulated by epigenetic mechanisms, such as histone deacetylation, DNA methylation, and polycomb repressive complex 2 (PRC2)-mediated histone 3 lysine 27 trimethylation (H3K27me3), which help limit the expression of MHC-I. In consistency, administration of the inhibitors for these processes, including DNA Methyltransferases inhibitor (DNMTi) [16] and Histone methyltransferase [17, 18] could up-regulate the expression of MHC-I complex. Non-coding RNA (ncRNA) is the other important part of epigenetic modulators, which plays major roles in regulating the expression of MHC-I. Hu et al. identified that miRNA 34a (miR34a) is enriched in abundance in the developing hippocampal neurons, which targets at the 3′UTR site of MHC-I mRNA, thus decreasing the expression of MHC-I and facilitating the normal development of neural morphology in developing hippocampal neurons [19]. Notably, in the context of cancer, miRNAs also participate in the regulation of MHC-I expression, which subsequently influences the process of anti-cancer immunity. Zheng et al. found that miR-148-3p serves as an oncogene by targeting on calnexin pathway, which suppresses the surface expression of MHC-I on tumor cells and inhibits the CD8^+^ T cell-dominated anticancer immunity in colorectal cancer [20]. Similar results were also demonstrated in esophageal adenocarcinoma, in which miR-148-3p and miR-125a reduce the expression of MHC-I and significantly shorten the overall survival of cancer patients [21].

In all, both genetic and epigenetic factors modulate the normal expressive mode of MHC-I under normal physiological conditions and any disturbance or imbalance within this regulatory network would lead to the impaired antigen presentation system and the subsequent pathological results.

**Fig. 1:**
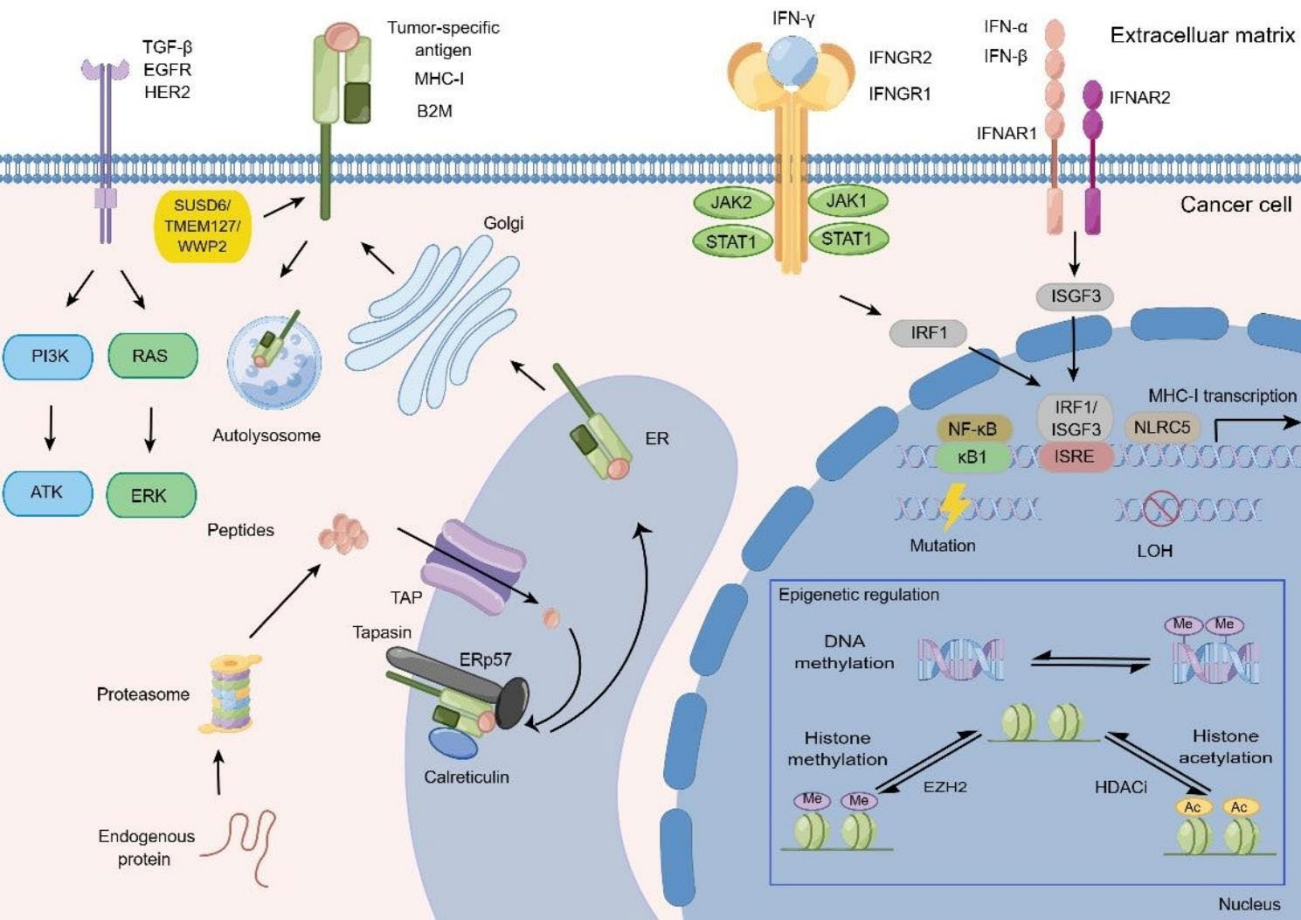
The modulation of MHC-I molecules-related antigenic processing and presentation mechanisms in cancer cell

MHC-I molecules composed of the heavy chain, light chain B2M, and tumor-specific neoantigen are presented on the surface. To create pMHC-I, endogenous proteins are processed in the proteasome, endoplasmic reticulum, and golgi. At the genetic level, gene alterations in MHC-I molecules that result in function loss mostly include mutations and LOH. At the transcriptional level, NLRC5 is a prominent transactivator of expression of MHC-I molecules. Reverse epigenetic modification in the MHC-I molecules alter the expression of MHC-I molecules, DNA methylation, histone methylation, and histone deacetylation are the most dominant modes of epigenetic regulation. IFN-α and IFN-β signaling dysregulation impacts the normal transcription of MHC-I molecules. IFN-γ is a frequent signaling pathway to regulate the MHC-I gene transcription. Other signaling pathways, including TGF-β, EGFR and HER2, can also act as regulators in the MHC-I mediated the antigenic presentation. In addition, cancer cells can hijack lysosome to degrade MHC-I molecules.

## The interactions between MHC-I and tumor microenvironment

The tumor microenvironment (TME) is an intricate ecosystem composed of cancer cells, immune cells, endothelial cells, and cancer-associated fibroblasts, as well as non-cellular components [22]. TME cells perform complicated tumor suppressive or supporting functions rather than acting as bystanders of tumorigenesis. In the TME, tumor immune surveillance is an effective strategy to recognize and eliminate emerging tumor cells. The basic structure and function of MHC-I determines that it is a key initiation step of the tumor immune response. This strategy is based on specific antigen presentation within tumor cells by MHC-I molecules [23]. In fact, there are myriad mechanisms by which immune surveillance might be evaded and rendered ineffective, such as impaired tumor-specific antigen presentation or trouble identifying pMHC-I. Here, we focus on the recent advances in how MHC-I interacts with TME cells and MHC-I-based immune surveillance and evasion.

### CD8^+^ T cells

T-cell-based acquired immune status is one of the most critical elements affecting tumor immune escape and immunotherapy efficacy. In TME, a key first step in the CD8^+^ T cell anti-tumor immune response is MHC-I-mediated antigen presentation and recognition. In principle, T cells have activating TCRs that can be triggered by their ligands, the pMHC-I complex. When pMHC-I presents a “non-self” peptide with an abnormally altered structure, the CD8^+^ T cells are programmed to attack or eliminate the aberrant cells, especially malignant cells and infected cells. Some cancers have impaired antigen processing and presentation mechanisms and insufficient expression of specific antigens to initiate T cell recognition. Impeding the interaction between the TCR and MHC-I molecules can negatively affect the anti-cancer response. Jongsma et al. reported that a lack of SPPL3 enhances the activity of the B3GNT5 enzyme and generates GSL which blocks MHC-I from communicating with the immune cells, inhibiting immune recognition [24].

Downregulation of MHC-I molecules, including classical MHC-I and B2M, can be seen in varied cancer [8]. To overcome the resistance in MHC-I-deficient cancer cells, Ito et al. identified alternative pathways for killing MHC-I-deficient cancer cells. One strategy is to target the TNF signaling and autophagy pathways, which cause tumor cells to die as a result of the cytokines released by T cells. Cross-presenting MHC-I-deficient cancer cell antigens by DC cells stimulated T cell infiltration and the generation of IFN-γ and TNF-α production [25]. In head and neck cancer cells, the chemokine CXCL14 enhances tumoral infiltration of CD8^+^ T cells, which trigger the generation of IFN-γ and TNF-α and restore the expression of MHC-I on tumor cells [26].

On both T cells and NK cells, NKG2A and CD94 dimerize to create an inhibitory receptor. ITIM is phosphorylated as a result of the contact between T cells and peptide-loading HLA-E, which transmits an inhibitory signal. It has been reported that NKG2A and HLA-E are upregulated in various cancer cells [27]. Hamind et al. demonstrated that CD94/NKG2A presence on T cells can impair IFNγ-mediated response. Enriched HLA-E:CD94/NKG2A interactions can suppress T cell activity [28]. Consistently, interruption of HLA-E and NKG2A interaction might enhance the clinical response by potentiating CD8^+^ T cell immunity. Additionally, they described negative feedback suggesting that HLA-E on tumor cells and its receptors on CD8^+^ T cells can be induced via cancer vaccines [29].

In short, MHC-I molecules are starting factors in the adaptive immune system. Tumor cells that lose surface MHC-I molecules can acquire the capability to escape from CD8^+^ T cell surveillance. Targeting this mechanism might be a promising strategy for clinical intervention. However, the common belief that downregulation of MHC-I constitutes an efficient strategy of immune evasion is challenged by the study that CD8^+^ T cells maintain the capacity to kill tumor cells even after MHC-I is lost. This is accomplished through interactions between T cell NKG2D and tumor NKG2D ligands [30]. Thus, further investigations into the relationship between MHC-I and CD8^+^ T cells in TME are necessary.

### Natural killer (NK) cells

The interaction between MHC-I and TCR is crucial for the activation of immunity. However, rather than having a single immune activating effect on the progression of tumors, MHC-I appears to have a complicated tale. Recent years have seen a rise in interest in the MHC-I-mediated immunosuppressive mechanism in TME. The NK cell is a vivid illustration.

Killer cell immunoglobulin-like receptor (KIR), a classical MHC-I specific inhibitory receptor localized on the surface of NK cells. Through the interaction of KIR with MHC-I molecules, normal cells containing MHC-I molecules inhibit the activation of NK cells. On the other hand, cancer cells lacking the MHC-I molecule, which leads to the loss of the MHC-I-KIR interaction, can activate NK cells. This process is called ‘missing-self recognition’. Owing to this property, NK cell-based immune therapy is a promising strategy for MHC-I deficient tumors [22]. Another inhibitory receptor is NKG2A. In microsatellite instable (MSI) cancer, HLA-E/B2M are aberrantly overexpressed and are associated with NKG2A-expressing CD94^+^ T cells and NK cells [31]. This result demonstrated that HLA-E:NKG2A plays a prompt role in immune evasion, it would seem to be a strong target for immune checkpoint blockade. Liu et al. uncovered that hijacking the immune checkpoint HLA-E:NKG2A can prompt circulating cancer cells to escape from NK cells [32]. Their results also imply that blockade of HLA-E:CD94-NKG2A may be a potential approach for cancer treatment. HLA-G can interact with its receptors ILT2 and KIR2DL4 on the NK cells, which leads to suppression of NK cell activity [33]. In addition, there are other activating receptors in NK cells. An activating receptor called NKG2D, recognizing ligands including MHC-I polypeptide-related sequence (MIC) and several UL16-binding proteins (ULBPs), is best studied. It is reported that continuous stimulation of activating receptors on the surface of NK cells causes desensitization. The shed ligands MICA and MICB from the tumor cells directly pair with the NKG2D, leading to NK cell desensitization [34]. In order to reestablish NK cell-mediated tumor immunity, Andrade et al. created an antibody to prevent the loss of MICA and MICB from cancer cells [35].

Moreover, the interaction of small-molecule components with MHC-I is important for shaping NK cells. Previous evidence uncovered that NK cells infiltrating MHC-I-deficient tumors acquired a hyporesponsive state, whereas others infiltrating MHC-I-sufficient tumor cells failed to induce an anergic state. The dysfunctional state can be reversed by inflammatory cytokines [36]. Recent studies have demonstrated that the tumor microenvironment alters the cytokines produced by NK cells, leading to changes in tumor progression. NK cells pre-exposed to tumor cells expressing MHC-I promote host extramedullary myelopoiesis, which is partly related to TNF-α secretion by tumor-experience NK cells [37].

Lastly, the ‘missing-self recognition’ by NK cell surveillance may be circumvented by tumors. Current NK-cell-based immune therapies have focused on the interaction between NK cells and tumor cells. Understanding the features of the tumor is essential to maximizing the NK cells’ untapped potential and developing clinical therapeutic interventions.

### Tumor-associated macrophages (TAMs)

TAMs have been identified as a key intertumoral regulator with pro- and anti-tumorigenic dual functions. TAMs are highly complex and plastic immune cells in the TME. It can be divided into M1 and M2 subtypes after activation. The M1 subtype is a classical anti-tumor killer cell, whereas the M2 subtype is an immunosuppressive subtype that expresses a range of suppressive cytokines. The structural molecules of the MHC-I complex, the heavy chain and B2M, can interact with TAMs to exert immunosuppressive effects.

A review summarized the roles of heavy chain for TAMs. The HLA-A, B, C in cancer cells suppress macrophage activation or stimulate alternative macrophage differentiation via the leukocyte lg-like receptors (LILRs) family. They also reported that the heavy chain in TAMs conducts the immunosuppressive function through inhibiting NK and T cell activities or releasing cytokines [38]. Similarly, the activation of macrophages can be adversely regulated by MHC-I light chain B2M. According to Li et al., B2M promotes the M2 phenotype in TAMs. Furthermore, they confirmed that M2 polarization is attributed to the B2M-induced TGF-β activation of the PI3K/ATK signaling in TAMs [39]. Recently, Barkal et al. elucidated that MHC-I can directly inhibit the phagocytic activity of TAMs. B2M of the MHC-I molecules can combine with the inhibitory receptor LILRB1 on the surface of macrophages, protecting cancer cells from phagocytosis [40].

These researches provided new insights into the function of MHC-I in the intrinsic immunity system. MHC-I molecules have the potential to reprogram TME into a tumor-promoting state. To disturb the macrophage-mediated tumor cell killing effect, MHC-I molecules can directly interact with immune cells or secrete immunosuppressive cytokines indirectly.

### Other cells

Dendritic cells (DCs) are well-known antigen-presenting cells in the immune response. The theory of antigen cross-presentation, once it was put forward, contributed to a deeper comprehension of DCs in the TME. Cross-presentation is a critical process by which DCs mediate antigen presentation in non-DC infections and activate CD8^+^ T cells. DCs take up tumor cells that are perishing and go through a maturation process. The antigens are processed and loaded onto MHC-I and MHC-II for presentation to CD8^+^ T cells and CD4^+^ T cells, respectively, as they migrate to the lymph node [41]. It is disappointing to note that DCs in TME might exhibit cross-presentation abnormalities that can be attributed to oxidized lipid. It precluded cross-presentation by the interaction between ox-tr-LB and the chaperone HSP70, which led to the translocation of the MHC-I to endosomes rather than the cell surface [42]. In fact, further research is still required to determine the processes underlying immune tolerance or lymph node metastases linked to DC cells.

Myeloid-derived suppressor cells (MDSCs) are well-known as immunosuppressive participants in TME [43]. MDSC has been discovered to nitrate a specific site of TCR/CD8 structure through cell-to-cell contact. This study demonstrated the T cell tolerance resulting from blocked communication between TCR and pMHC-I [44].

Regulatory T cells (Treg cells) are one of the most immunosuppressive cell populations in the T cell population. NK cell activity can be downregulated by Treg cells by interacting with NKG2D [22].

In conclusion, the interaction between MHC-I and immune cells plays a complex dual role in the tumor microenvironment, regardless of whether the tumor is progressing or being treated. In other words, it has the ability to activate acquired immune pathways and exert anti-tumor response, as well as suppress intrinsic immunity and promote the occurrence of immune escape. The immune status of the malignancy and the efficacy of the immunotherapy are ultimately determined by the coordinated action of multiple immune cells.

**Fig. 2:**
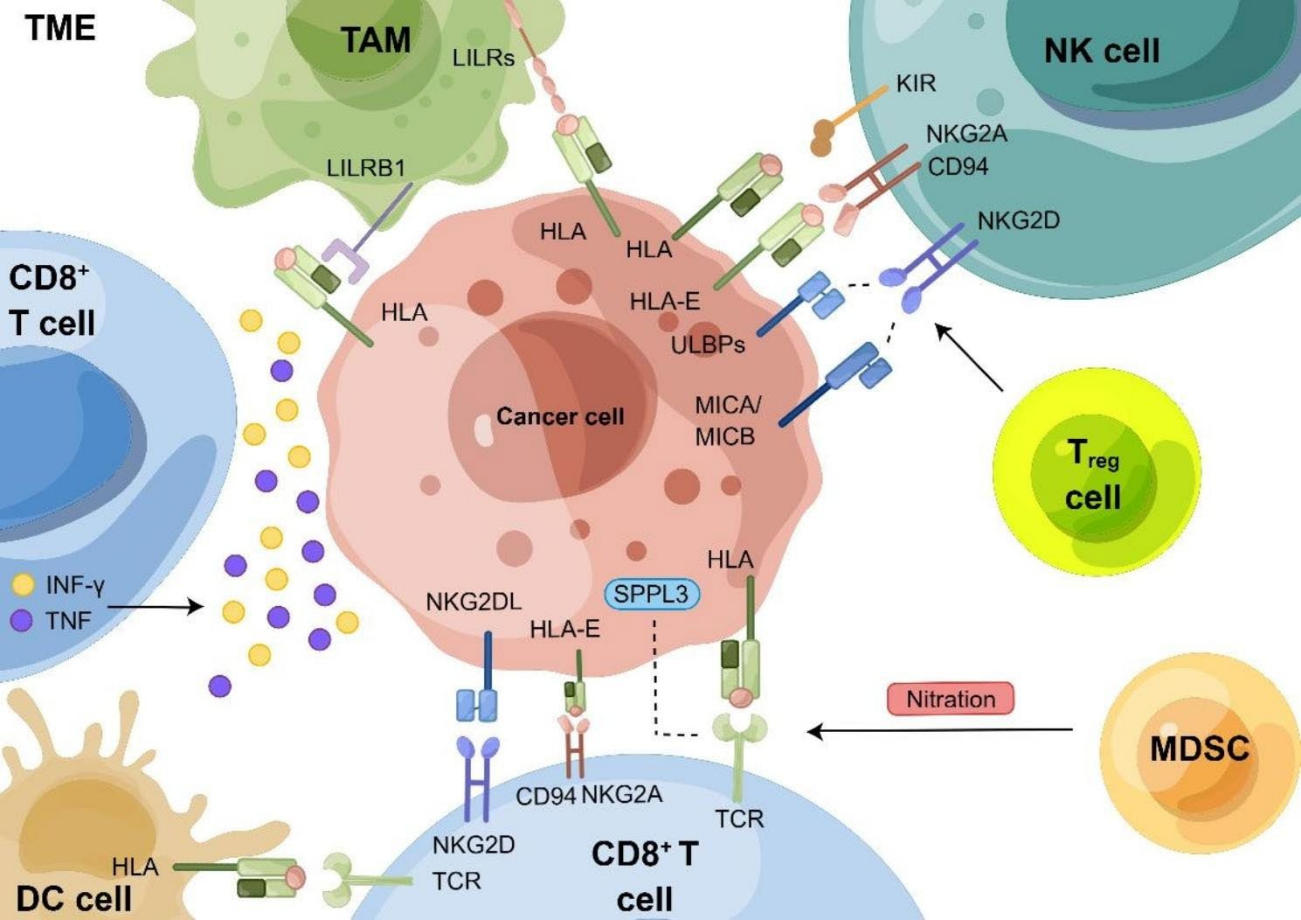
The interaction between the cancer cell and immune cells in tumor microenvironment

A complicated interaction network among the participants in the tumor microenvironment targets MHC-I. CD8^+^ T cells recognize pMHC-I on the surface of tumor cells via the TCRs. MDSCs can deactivate CD8^+^ cells through nitration of the TCR. Absent SPPL3 upregulates the cell surface GSL, which impedes the interactions with TCR. Both CD8^+^ T cells and NK cells have the inhibitory receptor CD94/NKG2A that combines with the HLA-E on the cancer cell. CD8^+^ T cells can release the various cytokines including INF-γ and TNF, and induce signaling activation. TAMs can shield tumor cells from phagocytosis by combining with B2M through LILRB1. KIR, an inhibitory receptor on NK cells, can bind to the HLA-A, B, and C. ULBPs, MICA/MICB, and Treg cells can all regulate NK cell activity via NKG2D. When tumor cells lack MHC-I molecules, DC cells can cross-present the tumor-specific antigen to CD8^+^ T cells.

## MHC-I and its component-related immunosuppressive functions

MHC-I is a double-edged sword for tumor regulation. In principle, the MHC-I-mediated antigen processing and presentation pathway transduces the tumor-associated antigens to CD8^+^ T cells via TCR, activating the acquired immune system. Also, MHC-I-deficient cells fail to transduce the inhibitory signal to NK cells and TAMs, resulting in the activation of the innate immunity system. Based on these MHC-I-related immune functions, tumor cells can exploit MHC-I to achieve immunosuppression. On the one hand, cancer cells can exploit complex regulatory networks including diverse genetic alternations, regulators, degradation, stress, and hypoxia to block pMHC-I expression. Tumor cells that lost or downregulated the expression of MHC-I fled from CD8^+^ T cell surveillance. On the other hand, overexpressed MHC-I molecules can interact with NK cells and TAMs and release the “Don’t eat me” signal to inactivate the innate immune system, leading to immunosuppressive functions.

### Genetic dysregulation

Genetic level modifications are the common strategy of tumor cells to impair the gene expression and function. MHC-I molecule mutations can affect the antigen presentation, thereby facilitating tumor cells escape from the control of immune surveillance. For example, 90% of cervical cancers present HLA-A, B, C and B2M mutations and lose the function of these genes, thus inducing resistance to T cell-based immunotherapy [45]. A pan-cancer analysis of MHC-I mutations was conducted based on gene expression in the TCGA database. They identified the mutation as being enriched in numerous cancer types [46]. MSI cancer is derived from defective DNA mismatch repair, alterations in the MHC-I gene are preferred in this cancer. A striking example was observed in the research in which MSI cancer patients tend to have a higher mutation frequency in the B2M gene [47, 48]. The MHC-I gene is polymorphic, which determines its ability to present multiple antigens. LOH is the most common alternation in classical MHC-I molecules and B2M. This means that either parental haplotype is lost, and neoantigens presented by the haplotype may be unable to present. This LOH may lead to immune escape, which is vulnerable to microenvironmental selection pressures in tumor evolution [49]. In patients with advanced melanoma, 29.4% of the B2M gene exhibited deletion and LOH deficiency [50]. Another sample is that HLA LOH occurs in 40% of non-small-cell lung cancer (NSCLC) [49]. We think that this mechanism is very cunning, it creates difficulties in T-cell recognition, and the repertoire of peptides presented by the excess haplotype has the potential to flee from the NK-cell cytotoxicity, but it requires experiments to verify.

### Epigenetic dysregulation

Tumorigenesis develops as a highly evolutionary and dynamic progression process in which epigenetics is also involved in the regulation of the expression of a variety of oncogenes. At the transcriptional level, MHC-I-related epigenetic dysregulation in cancer cells is a critical factor in immunosuppression. The reversibility of the epigenetic mechanism makes it a promising modality for regulation and possibly amenable to therapeutic intervention.

In mammalian genomes, DNA promoter methylation is a frequent and effective epigenetic regulation. Hypermethylation at HLA-A, B, C loci was found in varied cancer patients, including gastric cancer [51], esophageal squamous cell carcinoma [52] and cervical cancer [53]. In patients with MSI colorectal cancer, promoter methylation at the B2M gene was observed [54]. These studies demonstrated that transcriptional inactivation and downregulation of MHC-I molecules expression were associated with promoter hypermethylation. Therefore, we can conclude that DNA methyltransferase inhibitors may be an effective clinical intervention to enhance the response to immunotherapy. Advanced research reported that DNA methyltransferase inhibition upregulated the expression of MHC-I in various cancer types [55, 56]. Histone methylation is also a common methylation approach to achieve immune resistance and immune inhibition. Burr et al. reported that PCR2 mediates MHC-I gene silence via repressive H3K27me3 histone modifications. Enhancer of zeste homolog 2 (EZH2) is a facultative methyltransferase subunit of PRC2. Moreover, they also discovered that targeted EZH2 inhibition can reverse anti-tumor immunity [57]. Zhou et al. demonstrated that EZH2 can regulate the B2M promoter region via H3K27me3 [58]. They also revealed that combination EZH2 inhibition and anti-PD-1 therapy suppressed tumor progression. With the rapid development of bioinformatics, more in-depth analysis was conducted for MHC-I molecules. An encouraging study developed a CRISPR-based approach to accomplish targeted haplotype-resolved assembly of the MHC region. They successfully established an accurate analysis of the expression of MHC region alleles and DNA methylation modifications [59]. This approach can open up new avenues to elucidate immunosuppression based on MHC-I molecules.

Another typical epigenetic regulation in the control of MHC-I transcription is achieved through histone deacetylation. Previous studies have proved that histone deacetylase inhibitor (HDACi) treatment can enhance the expression of multiple antigen processing and presentation pathway-related genes, including MHC-I molecules, in melanoma cells [60]. As the research progressed, it was shown that HDACi upregulate MHC-I, which enhances antitumor immunity and facilitates the infiltration of cytotoxic T cells [18, 55, 56, 61–63]. In the clinical reports, HDACi may help MCC patients overcome MHC-I downregulation to address the drug resistance of anti-PD-1/PDL1 antibodies [64]. These HDACi-related findings lead us to the conclusion that histone deacetylation for MHC-I is an immunosuppressive process. Nevertheless, the underlying mechanisms are not yet clearly understood.

### Immune regulatory networks

Numerous studies confirm that MHC-I and its components are regulated by intricate oncogenic networks. IFN-γ is the core of MHC-I-mediated anti-tumor immunity. IFN-γ binds to its receptor, stimulates the following JAK/STAT signaling pathway, and induces MHC-I molecule production [22, 65]. The study reported that mutations were detected in IFN-γ/JAK/STAT signaling for varied cancer types. These deficiencies lead to immunosuppression of antigen presentation defects and resistance to ICB therapies [66, 67]. Mechanically, impaired IFN-γ/JAK/STAT signaling axis can inactivate histone dimethyltransferase WHSC1, leading to limited expression of MHC-I [17]. Besides, the combination of oxygen and glucose deprivation also resulted in decreased expression of the IFN signaling pathway and even failure to induce MHC-I molecules [68]. The RAS, MEK, ERK, and so on signaling cascades that make up the MAPK signaling pathway can be triggered by upstream HER2 and EGFR. In different cancer types, MAPK signaling is proven to be an MHC-I negative regulator [69, 70]. Inhibiting the MAPK signaling pathway can result in pMHC-I molecules target recognition by T cells. PI3K/AKT pathway is also dysregulated in the tumor microenvironment. Sivaram et al. have shown an inverse relationship between the PI3K/AKT pathway and MHCI expression in pancreatic cancer, leading to immune evasion [71]. A similar result has been found in human head and neck squamous cell carcinomas [72]. The Wnt pathway also downregulates the expression of MHC-I molecules in glioma stem cells [73]. The HLA-A mRNA-binding protein MEX3B can mediate resistance to ICB by downregulating HLA-A on the surface of melanoma cells [74]. On the other hand, non-coding RNA can also inhibit the expression of the antigen presentation pathway. Molecular chaperone calnexin and MHC-I expression can be suppressed by miR-148a-3p in colorectal cancer, attenuating tumor growth [20]. Colangelo et al. reported that calreticulin can be a target of miR-27a, repressing the surface MHC-I antigen display [75].

Tumor cells use a variety of strategies to control the immune system, which involves a complex regulatory network, in order to suppress the immune response. Targeting the inhibition of MHC expression at multiple levels in order to prevent it from interacting with T cells is one of the main areas of research. However, MHC-I in intrinsic immunity also serves a crucial function for NK cells and TAMs, but there is a lack of relevant research on how different regulatory molecules interact with them.

### MHC-I degradation

The autophagy pathway is one strategy to regulate surface MHC-I expression. Cancer cells promote the redirection of MHC-I molecules to lysosome-mediated degradation. This immunosuppressive mechanism can prevent tumor cells from being recognized by CD8^+^ T cells. A recent study published in *Nature* proposed that autophagy can degrade MHC-I molecules, which results in pancreatic cancer cells escaping CD8^+^ T cell-mediated immune surveillance. This immune evasion can be reversed by genetically or pharmacologically inhibiting autophagy with chloroquine [7]. Yamamoto et al. revealed that MHC-I molecules will enter lysosomal degradation through the cargo receptor NBR1-mediated autophagy process in pancreatic ductal adenocarcinoma (PDAC) cells. It leads to reduced MHC-I expression on the cell surface and impaired immunotherapy [7]. Similarly, Fang et al. discovered that the interaction between MAL2 and MHC-I molecules and RAB proteins, that is, endosome-related proteins, converts to lysosomal degradation in breast cancer cells [76]. Thus, it inhibited tumor antigen presentation and decreased CD8^+^ T cell infiltration. They also observed that MAL2 deletion can significantly enhance the infiltration of CD8^+^ T cells in the preclinical model. Consistently, suppression of PGRN restores MHC-I expression in PDAC cells by decreasing lysosomal activity and autophagosome degradation [77]. Notably, the authors constructed a PDAC mouse model using a model antigen, LCMV-GP33. Tumors with LCMV-GP33 are sensitized to gp33-TCR transgenic T cells, regaining tumor immunogenicity and tumor antigen-specific cytotoxicity. YTHDF1 deficiency constrains lysosomal-related proteolysis of MHC-I molecules. In order to target YTHDF1 in vivo, Lin et al. developed a system for exosome-mediated CRISPR/Cas9 delivery, which leads to YTHDF1 depletion and restores tumor immune surveillance [78]. These studies persuaded us that decreasing CD8^+^ T cell infiltration and immunosuppression may occur by exploiting the autophagy pathway. Additionally, we can speculate that targeting this process can restore tumor immune surveillance and make patients more sensitive to ICB therapy.

### Immune cells

Classical MHC-I-related immunoregulatory mechanisms include the genetic, transcriptional, translational, and post-translational levels. A series of findings suggested that cancer cells will directly suppress the expression of MHC-I molecules or interfere with the molecules involved in the antigen processing and presentation pathway. It impairs the CD8^+^ T cell-mediated immune response and flees from immune surveillance. In addition to directly or indirectly affecting T cells, the suppressive effect of MHC-I on the intrinsic immune system should not be underestimated.

MHC-I can exert its immunosuppressive function through a variety of immune cells. For example, this can be achieved by engaging with the inhibitory receptor KIR on NK cells or by targeting macrophages through various inhibitory pathways. In addition, the inhibitory cells Tregs and MDSCs suppress the cytotoxicity of NK cells and T cells, respectively. In the previous section, we offered a more extensive review.

## The association between MHC-I molecules and pathological characteristics of human cancers

The dual function of MHC-I molecules in tumor immunity leads to different immune states in patients, which will affect the growth and metastasis of tumor cells and eventually influence the pathological characteristics of human cancers as well as the prognosis of patients. Therefore, MHC-I molecules might be utilized to establish the molecular stage of cancer, and have great application potential as biomarkers or models to predict tumor diagnosis, prognosis, recurrence, and immunotherapy response.

Traditionally, it is believed that cancer cells with downregulated or deficient surface MHC-I molecules are more likely to escape T cell surveillance, thus leading to a poor prognosis. Studies revealed classical MHC-I molecules and B2M may be an immunological prognostic factor for varied cancers [79–83]. As for immunotherapy, downregulation of classical MHC-I has been identified as a risk factor for recurrence in bladder cancer patients treated with BCG immunotherapy [84]. Zhao et al. revealed that a high level of B2M expression could enhance the anticancer immune response [85]. Other studies reported the opposite results due to the dual function of MHC-I molecules. They considered that cancer cells lacking MHC-I molecules were more sensitive to NK cells. This may be the reason for the good prognosis of low-level expression of HLA [86, 87]. Intriguingly, Watson demonstrated that the high expression or absence of HLA correlates with a better prognosis than the low expression of HLA in colorectal cancer [88]. In summary, CD8^+^ T cells can attack cancer cells with high HLA expression, whereas TAMs and NK cells can eliminate cancer cells without MHC-I expression. Low expression of MHC-I might escape both acquired and inherent immunity. We speculate that the reason for the opposite prognostic results may be relative expression. Another possibility is that the heterogeneity also causes different tumor microenvironment, ultimately leading to different prognostic outcomes for patients.

As for non-classical MHC-I, it is usually considered to be upregulated in cancer. Physiologically, a reasonable explanation is that nonclassical MHC-I molecules play an immune tolerance role to protect embryos from maternal immune system attack [89], cancers may exploit this property for immune escape. HLA-E [90–92], HLA-F [93–95] and HLA-G [96–98], which have been identified as novel immune checkpoints, can bind to the inhibitory receptor of immune cells to induce immune tolerance and are regarded as prognostic factors. Morinaga et al. reported that the combination of HLA-E expression and NK status can be a more sensitive prognostic biomarker in advanced gastric cancer [90]. HLA-G is a significant prognostic indicator of colorectal cancer, and the possible mechanism is the binding of HLA-G to its suppressive immune checkpoints ILT-2 and ILT-4 [96, 98, 99]. Wu et al. have revealed that high HLA-F expression is closely associated with local recurrence and distant metastasis of nasopharyngeal carcinoma [93]. A study reported that HLA-G is a biomarker of tumor cell susceptibility to therapeutic agents by the immune response or treatment [97].

With the rapid development of bioinformatics, numerous hub genes and prognostic models were identified based on the MHC-I-related genes [82, 100], it showed great significance for prognostic prediction and guiding immunotherapy. These results inspired us to believe that the impact of MHC-I molecules on pathological characteristics varies considerably by cancer. Deeper mechanisms and larger cohorts should be enrolled to determine the pathological characteristics of MHC-I molecules in different cancers.

**Table 1:** Function of human cancer MHC-I expression

Cancer type	MHC-I type	Number of patients	Analysis method	Function	Reference
Muscle-invasive bladder cancer	Pan HLA-I (HLA-A, B, C)	65	Immunohistochemistry by anti-pan HLA-I antibody EMR 8 − 5	Positive	[79]
	Pan HLA-I (HLA-A, B, C)	30	Immunohistochemistry by anti-pan HLA-I antibody EMR 8 − 5	Positive	[84]
Non-small-cell lung cancer	B2M	/	Bioinformatic analysis	Positive	[85]
Clear cell renal cell carcinoma	Pan HLA-I (HLA-A, B, C)	45	Immunohistochemistry by anti-pan HLA-I antibody EMR 8 − 5	Positive	[80]
Invasive breast cancer	Pan HLA-I (HLA-A, B, C)	439	Immunohistochemistry by anti-HLA-I heavy chain antibody HC10	Negative	[86]
	B2M	424	Immunohistochemistry by polyclonal rabbit-anti-B2M antibody	Negative	[86]
Advanced gastric cancer	HLA-E	232	Immunohistochemistry by anti-HLA-E antibody MEM-E/02	Negative	[90]
Nasopharyngeal carcinoma	HLA-F	74	Immunohistochemistry by anti-HLA-F antibody EPR6803	Negative	[93]
Colorectal cancer	Pan HLA-I (HLA-A, B, C)	97	Immunohistochemistry by anti-pan HLA-I antibody EMR 8 − 5	Positive	[81]
	Pan HLA-I (HLA-A, B, C)	455	Immunohistochemistry by anti-HLA-I heavy chain antibody HC10	High expression and absent lead to a good prognosis, low expression leads to a poor prognosis	[88]
	HLA-B/C	2863	Bioinformatic analysis through StataSE	Positive	[101]
	HLA-A	88	Immunohistochemistry by anti-HLA-A antibody HCA2	Negative	[102]
	HLA-G	157	Immunohistochemistry by anti-HLA-G-PE antibody MEM-G/09	Negative	[96]
	HLA-G	137	Immunohistochemistry by anti-HLA-G antibody MEM-G/2	Negative	[98]
	HLA-E	137	Immunohistochemistry by anti-HLA-E antibody MEM-E/02	Positive	[98]
	HLA-G	201	Immunohistochemistry by anti-HLA-G antibody HGY	Negative	[99]
Endometrial cancer	Pan HLA-I (HLA-A, B, C)	554	Immunohistochemistry by anti-HLA-I heavy chain antibody HC10	Positive	[103]
Ovarian cancer	Co-expression of HLA-B/C and B2M	232	Immunohistochemistry by anti-HLA-I heavy chain antibody HC10 and polyclonal anti-B2M antibody	Positive	[104]

Positive, high expression of MHC-I leads to a favorable prognosis or low expression leads to a poor prognosis. Negative, high expression of MHC-I leads to a poor prognosis or low expression leads to a favorable prognosis

## Therapeutic strategies and advances targeted on MHC-I

### Immunotherapy

#### Immune checkpoint blockade

ICB therapy is regarded as a milestone in cancer immunotherapy, particularly the anti-CTLA4, anti-PD-1, and anti-PD-L1 drugs. Though ICB therapy has maintained tumor shrinkage and prolonged life expectancy in some patients, non-response and resistance remain barriers to treatment. Numerous mechanisms have been investigated, with the antigen presentation defect being a key one.

Recently, HLA-E:CD94/NKG2A has been identified as a novel immune checkpoint. Targeting this immune checkpoint can induce the anti-tumor activity of CD8^+^ T cells and NK cells [27, 105]. The most promising anti-NKG2A mAb, called monalizumab, prevents the interaction between NKG2A and HLA-E and exhibits a potent therapeutic impact on several cancer types, such as NSCLC, CRC, and SCCHN [106]. Disappointingly, the highly anticipated monalizumab for head and neck cancer finally failed in the Phase III clinical trial in August 2022. Its clinical therapeutic effect in other cancers still requires further clinical trials to validate.

Alteration of genes that encoding MHC-I molecules will affect ICB efficacy. Previous research documented LOH at HLA in resistant tumor that are treated with T cell transfer therapy [107]. Later, relevant clinical research has reported that HLA homozygosity and LOH in cancer patients represent a genetic barrier to effective ICB therapy [108]. A similar outcome could be demonstrated in B2M, namely that B2M LOH is prevalent in cancer patients who have a poor response to or non-response to ICB therapy [50, 109]. In addition, genetic mutations in B2M, resulting in the formation of defective HLA, may also be associated with acquired resistance to ICB treatment [110, 111].

Impaired antigen presentation may be a mechanism of resistance to ICB therapy, including HLA heavy chain, light chain B2M, and other antigen presentation components [110, 112, 113]. This is associated with increased presentation of tumor antigen as well as T cell recognition. Therefore, upregulation of MHC-I molecules expression is a promising therapeutic strategy to enhance the synergy with ICB. PCSK9 inhibition potentiates MHC-I expression, which promotes T cells to infiltrate the tissue, making the tumor more sensitive to the immune checkpoints [114]. In order to conduct this test, Liu et al. used two anti-PCSK9 antibodies (evolocumab and alirocumab), which have been licensed for treating hypercholesterolemia. The combination of ICB and anti-PCSK9 antibody has to be tested in clinical use. Dysregulation of signaling pathways, such as interferon (IFN) pathway [113, 115–117], MAPK pathway [69], STAT pathway [117–119] and EGFR pathway [120], may result in aberrant MHC-I molecules production. In this condition, different molecules in the signaling pathway have been the targets of drugs. IFN-γ could induce the HLA expression [117]. Kang reported that the MEK inhibitor trametinib could block the MAPK pathway and thereby increase the MHC-I, which may lead to improved ICB efficacy [121]. In addition, bortezomib, a proteasome inhibitor (targeted at STAT1), can enhance the expression of HLA [118]. Lenvatinib restored STAT1 phosphorylation and the expression of B2M. When paired with an anti-PD-1 antibody, lenvatinib had stronger anticancer activity [119].

The expression of MHC-I molecules can be regulated epigenetically, typically by the use of epigenetic inhibitors, such as DNMTi [55, 56], HDACi [55, 62, 63] and EZH2 inhibitor [58]. Notably, Ugurel et al. reported 4 cases with non-response to ICB therapy enhanced HLA expression with the combination with HDACi [64]. Combining EZH2 inhibitior with ICB reduces tumor growth by changing the expression of B2M [58]. Therefore, we can conclude that these targeted drugs and epigenetic drugs restore MHC-I molecules expression and thus enhance their synergistic effect with ICB. It urged that further research be conducted to confirm the specific mechanisms.

Additionally, the effectiveness of ICB therapy will be enhanced by using it in combination with chemotherapy, radiotherapy, and other strategies due to the upregulation of HLA.

Abnormal expression of immune checkpoint molecules in human cancers has become the dominant mechanism by which cancer cells escape the immune response and ultimately lead to tumor development. With in-depth and extensive research, MHC-I molecules have been identified as novel immune checkpoint molecules [97]. There is an urgent need to explore the role of MHC-I molecules as immune checkpoints in ICB therapies. Perhaps medications that specifically target these immune checkpoints will become available in the future.

#### Cancer vaccines

Cancer vaccines, which can be classified as prophylactic and therapeutic vaccines, offer a promising way to improve the anti-tumor response. Since Alvaro Morales used Bacillus Calmette Guerin (BCG) to treat superficial bladder cancer in 1976, therapeutic vaccines have emerged [122]. Nowadays, BCG is still the standard treatment for non-muscle invasive bladder cancer. Besides, some research uncovered that HLA status plays a crucial role in the clinical characteristics of bladder cancer patients following BCG therapy [123–125].

Recently, some scientists conducted studies on personalized vaccines. This ushers in a new age of cancer vaccines and brings new research directions worth exploring. Targeting tumor neoantigens with high affinity for HLA has become acknowledged as feasible targets for personalized therapeutic vaccines. Tumor neoantigen vaccination can enhance the anti-tumor immune response, notably neoantigen-specific T cells [126–128]. Therapeutic vaccines have improved specificity and safety compared to conventional radiation and chemotherapy therapies.

In fact, the MHC-I genotype of patients has been shown to affect the clinical outcome [129–131]. Frameshift mutations in calreticulin occur in myeloproliferative neoplasm. However, MHC-I alleles that exhibit mutant neoepitopes with high affinity are underrepresented. They utilized a modified heteroclitic peptide vaccination method for patients to efficiently elicit T cell response [132]. Moreover, one of the possible causes of cancer vaccine resistance is abnormal MHC-I molecule expression. Cancer vaccination with ODN1862 adjuvant was only marginally effective in MHC-I-negative models that deleted the B2M gene in TC-1 cell line [133]. Cao et al. have developed a nanovaccine named HA-OVA-AuNPs that enhances proteasome activity and downstream MHC-I antigen presentation [134].

Neoantigen-specific T cells are limited in TME, expanding cytotoxic T lymphocytes (CTLs) against tumor cells may become a prospective strategy. Montfoort et al. revealed that therapeutic cancer vaccines targeting HLA-E:NKG2A might be a potential strategy to activate CD8^+^ T cell and NK cell immunity in microenvironment [29]. Intriguingly, some therapeutic cancer vaccines aim to disguise tumor cells as human CMV-infected cells by showing CMV-pMHC on their surface, allowing CMV-specific CTL to detect and lyse them [135, 136]. Through these strategies, bystander T cells [137] that recognize antigens unrelated to cancer were fully mobilized to attack tumor cells.

In addition, cancer vaccines combined with other therapy strategies aim to foster a favorable immune environment and enhance immune responses. Zhu et al. created the first engineered *Lactococcus lactis*-based cancer vaccine using probiotic loading neoantigens for ongoing activation [138]. Combining personalized cancer vaccines with PD-1, PD-L1, and CTLA4 inhibitors is now being used in different clinical trials to treat various cancer types [131]. An ICB immunotherapy, atezolizumab, and an individualized neoantigen vaccine with up to 20 MHC-I and MHC-II restricted neoantigens in nanoparticles autogene cevumeran are being tested in a phase I clinical trial [139].

#### Chimeric antigen receptor T cell (CAR-T) therapy

CAR-T therapy mediates non-MHC-restrictive cancer cell death by transducing CAR on the surface of T cells to detect and eliminate cancer cells.

Utilizing allogeneic CAR-T cells can be an efficient strategy to overcome the poor quality and quantity of autologous T cells. However, when HLA divergence occurs, the host immune system rejects allogeneic T cells. This obstacle can be solved by the CRISPR/Cas9 system to create negative HLA T cells [140]. Notably, negative MHC-I might activate NK cells, which then act as the main cause of CAR-T cell death [141]. Therefore, more studies are required to find out how to prevent them from killing CAR-T cells.

There are still studies being done on how to improve the efficiency of CAR-T cells. It has been demonstrated that INF-γ can be secreted by CAR-T cells, which will upregulate MHC molecules in cancer cells [142]. Moreover, combination therapy is a way to enhance the effect of CAR-T therapy. Radiation therapy can increase MHC-I expression, enabling T cells to more readily detect cancer cells. Therefore, combining CAR-T therapy and radiation therapy may have a synergistic impact [143, 144]. These findings provide an essential guide for the synergistic benefit of combining CAR-T therapy with conventional anticancer therapies targeted at MHC-I molecules.

#### Cytokine therapy

Cytokines have powerfully modulated functions in cell communication, affecting the tumor immune microenvironment and triggering anticancer responses. The majority of researchers believe that MHC-I-deficient cancer cells are killed by NK cells because they cannot bind inhibitory receptors on NK cells [145]. In fact, the cancer cells can bypass immune surveillance by NK cells, but cytokine therapy with IL-12 and IL-18, or H9 can reverse the anergic NK cells [36]. IFN molecules are promising cytokines targeted at MHC-I because they prompt DCs or T cells to eliminate cancer cells through upregulating MHC-I molecules [146]. Combination therapy with ICB has been done in clinical trials [147].

### Radiotherapy

Radiotherapy provides an effective therapeutic strategy to eliminate cancer cells. Biological mechanisms have been proven to include DNA damage response, immune modulation, and altering TME in cancer cells [148]. Notably, MHC-I could be upregulated by radiation, which makes it possible to improve antigen presentation and increase the recognition capacity and cytotoxicity of T cells [149–152].

Some researchers tried to demonstrate the complicated mechanism. Radiation activated IFN signaling, which enhanced the expression of MHC-I and antigen presentation, overcoming the resistance to ICB therapy [115]. In addition, radiation might induce the expression of NLRC5, impacting MHC-I expression and easing ICB limitation [151]. DNA damage has been proven to cause elevated MHC-I [153]. Whether the DNA damage response caused by radiotherapy still deserves further study and exploration.

Combination therapy of radiotherapy with other therapies can be an emerging strategy for treating cancer. Firstly, combined with adoptive T cell therapy, radiotherapy boosts the efficacy of the anticancer response by upregulating MHC-I expression and enhancing the sensitivity to cytotoxicity T cells [152]. Similarly, radiotherapy combined with GM-CSF vaccination is more effective in gliomas [154]. We can hypothesize that increasing MHC-I expression by radiotherapy may be the mechanism for synergistic effect with other immunotherapies but the underlying mechanisms require experiments to verify. On the other hand, radiotherapy can induce more neoantigen release and prompt T cell infiltration [128], which can circumvent the problem of insufficient neoantigen presentation and failure to activate the T cells. It indicates that radiotherapy can be combined with other therapies that can upregulate MHC-I. From this perspective, radiotherapy may be unsuitable for MHC-I-deficient cancers.

### Conventional chemotherapeutics and targeted agents

There is little research on how MHC-I is impacted by conventional chemotherapeutics and targeted agents. Enhancing MHC-I expression is the most common mechanism to promote anticancer responses in these therapies. Cisplatin, a DNA damaging agent, is widely used in clinical anticancer practice. Accumulating evidence points out that it has immunomodulatory effects and elevates MHC-I expression in different types of cancers [155], possibly related to the activation of IFN-β, NF-κB signaling pathway [116]. Moreover, cisplatin also upregulates MHC-I in antigen-presenting cells, such as DC cells [156]. Zhou et al. found that the lysine acetyl transferases CBP regulate MHC-I expression and neoantigen amounts in human cancer. DNA damaging drugs upregulate MHC-I dependent on activation of NF-κB [153]. Liu et al. reported that cyclophosphamide, oxaliplatin, and gemcitabine stimulate the expression of HLA by cancer cells [157]. Similar results were also found in gefitinib [158], MAPK inhibitors [159] and 5-Fluorouracil [160]. The study by Liu et al. similarly showed that PCSK9 Binds to pMHC-I to promote its degradation in lysosomes [114]. A solid foundation has been established for the introduction of PCSK9 inhibitors, a lipid-lowering medication with clinical approval, into oncology immunotherapy. They also show the evidences on targeting PCSK9 also improves the anti-tumor activity of PD-1 immune checkpoint. A study intriguingly suggests that downregulated MHC-I expression caused by imatinib is another way to boost immunity attribute to the relocation of NK cells to cancer [161]. In addition to altering the immunomodulatory effect by affecting MHC-I, chemotherapy can directly activate CD8^+^ T cells in an MHC-I-independent manner [162].

## Conclusions

In conclusion, MHC-I and its related molecules are crucial participators in the tumor microenvironment. Classical antigen presentation functions by MHC-I maintain the basic anticancer immunity of CD8^+^ T cells, which endows MHC-I with an indispensable place in the battle against tumor cells. Nevertheless, emerging studies generally uncovered the immune escape capabilities dominated by MHC-I in cancer. Multiple factors coherently modulate the expression and function of MHC-I in the context of cancer. MHC-I-related pathways may be a promising and efficacious target for synergizing with cancer immunotherapy. However, the identification and explanation of crucial molecules modulating the MHC-I functioning directions and their definite mechanisms are warranted. We hope this review would help summarize the latest and key knowledge of MHC-I in cancer, and, more importantly, inspire more studies on the therapeutic exploration based on MHC-I molecules.

### Electronic supplementary material

Below is the link to the electronic supplementary material.

Supplementary Material 1

## Data Availability

Not applicable.
