# Supplementary material for: Targeting MHC-I molecules for cancer: function, mechanism, and therapeutic prospects
Source: Mol Cancer. 2023 Dec 2;22:194. doi: 10.1186/s12943-023-01899-4 (PMC10693139; doi:10.1186/s12943-023-01899-4)
Supplement: Supplementary file 1 — Supplementary Material 1 [file 12943_2023_1899_MOESM1_ESM.pdf]

Abbreviations

MHC-I	Major histocompatibility class I
B2M	β2-microglobulin
TCRs	T-cell receptors
ICB	immune-checkpoint blockade
CAR	chimeric antigen receptor
HLA	Human Leukocyte Antigen
pMHC-I	peptide/MHC-I
ER	endoplasmic reticulum
TAP	Transporter associated with Antigen Processing
ERAP	ER aminopeptidases
NLRC5	Nucleotide-binding oligomerization domain-Like Receptor family Caspase recruitment domain containing 5
CITA	Class I Transactivator
ISRE	interferon- stimulated response element
PRC2	polycomb repressive complex 2
H3K27me3	histone 3 lysine 27 trimethylation
DNMTi	DNA Methyltransferases inhibitor
ncRNA	non-coding RNA
miR34a	miRNA 34a
LOH	loss of heterozygosity
TME	tumor microenvironment
GSL	glycosphingolipid
HNC	head and neck cancer
NKG2D	natural killer group 2D
NKG2DLs	tumor NKG2D ligands
NK	natural killer
KIR	killer cell immunoglobulin-like receptor
MSI	microsatellite instable
MIC	MHC-I polypeptide-related sequence
ULBPs	UL16-binding proteins
TAMs	tumor-associated macrophages
LILRs	leukocyte lg-like receptors
DCs	dendritic cells
MDSCs	myeloid-derived suppressor cells
Treg cells	regulatory T cells
NSCLC	non-small-cell lung cancer
EZH2	enhancer of zeste homolog 2
HDACi	histone deacetylase inhibitor
PDAC	pancreatic ductal adenocarcinoma

LCMV	lymphocytic choriomeningitis virus
GP	glycoprotein
IFN	interferon
BCG	Bacillus Calmette Guerin
MPNs	myeloproliferative neoplasms
CTLs	cytotoxic T lymphocytes
CMV	cytomegalovirus
CAR-T	chimeric antigen receptor T cell
APCs	antigen-presenting cells
CBP	p300/CREB binding protein
